# Medical expenditure and unmet need of the pre-elderly and the elderly according to job status in Korea: Are the elderly indeed most vulnerable?

**DOI:** 10.1371/journal.pone.0193676

**Published:** 2018-03-23

**Authors:** Hwa-Young Lee, Naoki Kondo, Juhwan Oh

**Affiliations:** 1 JW LEE Center for Global Medicine, Seoul National University College of Medicine, Seoul, Republic of Korea; 2 Takemi Program in International Health, Department of Global Health and Population, Harvard T.H.Chan School of Public Health, Boston, MA, United States of America; 3 Department of Health Education and Health Sociology, School of Public Health, The University of Tokyo, Tokyo, Japan; University of West London, UNITED KINGDOM

## Abstract

Increase in the elderly population and early retirement imposes immense economic burden on societies. Previous studies on the association between medical expenditure and working status in the elderly population have not adequately addressed reverse causality problem. In addition, the pre-elderly group has hardly been discussed in this regard. This study assessed possible causal association between employment status and medical expenditure as well as employment status and medical unmet needs in a representative sample of the Korean elderly (aged≧65) and the pre-elderly (aged ≧50 and < 65) adults from the Korea Health Panel Data (KHP). Dynamic panel Generalized Method of Moments (GMM) estimation was employed for the analysis of medical expenditure to address reverse causality, and fixed effect panel logistic regression was used for the analysis of unmet need. The results showed no significant association between job status and medical expenditure in the elderly, but a negative and significant influence on the level of medical expenditure in the pre-elderly. Unemployment was a significant determinant of lowering unmet need from lack of time while it was not associated with unmet need from financial burden in the fixed-effect panel model for both the elderly and pre-elderly groups. The pre-elderly adults were more likely to reduce necessary health service utilization due to unemployment compared to the elderly group because there is no proper financial safety net for the pre-elderly, which may cause non-adherence to treatment and therefore lead to negative health effects. The policy dialogue on safety net currently centers only on the elderly, but should be extended to the pre-elderly population.

## Introduction

Most of the developed countries are currently experiencing aging population due to low fertility rate and prolonged life expectancy. These demographic changes are expected to impose immense economic burden on societies since a disproportionate share of health resources is consumed by the elderly [1, 2]. In addition, aging is also a risk factor for financial insecurity and unmet health care utilization from the perspective of the individuals because opportunities to participate in labor market reduce in accordance to aging [3].

Economic activity has been persistently hypothesized to benefit health, thus reducing healthcare utilization and medical expenditure for the elderly [4, 5]. It is also argued that unemployment causes stressful situation [4, 6] and disconnections in social network, which leads to physical deterioration [7, 8]. Taken together, prior literatures suggest that unemployed individuals, who are less healthy and have more time on their hand, are likely to seek more healthcare services compared to the employed ones. However, previous studies supporting the health benefits of employment for the elderly have always been questioned on how well they have addressed the potential reverse causation; that is, the observed association may simply be an artifact of the elderly with better health remaining in economic activity longer [9–11]. This bidirectional association between employment and health care utilization challenges causal inference [12, 13]. In addition, because there are many factors that determine utilization of health service among the elderly, the pattern of unmet needs should be considered simultaneously to better understand whether the change in medical expenditure results from change in health status or other health-unrelated factors.

Another important population that contributes to increasing healthcare cost but rarely discussed in the literature is the pre-elderly group. Adults over age 50 start to face increasing medical need while they are getting vulnerable to job loss more and more due to consistently increasing unintentional early retirement. This situation put them at risk for financial distress without a proper safety net. In Korea, benefits of old age pension, which is the primary safety net, starts at the age of 65. Hence, it is extremely hard for the pre-elderly group (aged ≧50 and <65) to find financial resources when they lose job unless they suffer from disability.

This study addresses these gaps in the previous studies to shed light on a question of possible causation between employment status and medical expenditure among the elderly by using a more sophisticated methodologies to control for reverse causation using a longitudinal data from Korea [14, 15]. Additionally, we also investigate the change in unmet need according to the change in employment status. Finally, whether the same pattern in medical expenditure and unmet need appear in the pre-elderly group was also investigated.

## Materials and methods

### Data

This study used the Korea Health Panel Data (KHP) between 2008 and 2014 for the analysis of medical expenditure and between 2009 and 2014 for the analysis of unmet needs. The KHP includes information on socioeconomic status (SES), utilization of health service, medical expenditure, health status, and health behavior. The KHP employed stratified two-stage cluster sampling strategy for selecting households. All the household members within the selected households were investigated.

Data were collected mainly by face-to-face interview and also housekeeping books or receipts of healthcare spending to minimize recall bias. The household members sampled for the panel survey normally recorded the purpose of their medical visits and the total amount of payment they used in health facility or pharmacy and showed it when the investigators visited for survey interview. Therefore, data contain all information on out-of-pocket payment including payment for uninsured medical services and co-payment. We restricted our sample to individuals aged more than 65 years old for the elderly group and between 50 and 65 for the pre-elderly group.

### Outcome variable

The first outcome variable is medical expenditure per year, which included expenditure for emergency service, inpatient and outpatient services, transportation fee for emergency service (ambulance), and drug cost. The second outcome variable is unmet needs, a concept of necessity for medical service based on individual judgment. The participant was asked whether he/she experienced not being able to receive medical care such as consultation or examination despite their need, which was answered as “yes” or “no”. Moreover, reasons for not being able to seek medical care were defined as “unmet need due to financial matter” and “unmet need due to lack of time”[16].

### Independent variables

The main variable of interest is employment status measured by asking whether the respondent is working for living or not and constructed as a binary variable. Other covariates were chosen based on previous empirical analyses. Household characteristics such as household type (single, only couple, or couple with a child), house ownership (owner or rent), and household income quintiles were included. As individual characteristics, age (categorized in 5-year interval from 65 years for the elderly and from 50 to 64 years old for the pre-elderly), gender, and education (middle school graduate or less, high school graduate or less, more than university) were controlled. Insurance type was also included, in addition to income level, because of evidence on differences in healthcare utilization between two types of insurance even at the same income level [17]. Binary indicators for disability status and chronic disease status were also included as a proxy for medical demand.

### Statistical analyses

Medical expenditure data typically exhibit a few distinguished characteristics. First, they sometimes have a large mass of observations with zero cost. Secondly, a minority of extremely high-cost patients results in a skewed distribution to the right. One of the common approaches used to treat this problem is OLS regression with a positive shift at zero. However, the choice of the constant used to shift in this method is rather arbitrary. Another shortcoming of this method is that it requires re-transformation back to the original scale. Another possibility is to use the Tobit model based on the concept of latent variables [18–20]. However, this model is, effectively, a censored normal regression, which means that it’s sensitive to normality and heteroscedasticity assumption[21]. Two alternative approaches used in analyzing expenditure data with the least controversy are Generalized Linear Model (GLM) and Two-part model [21]. We employed GLM with gamma distribution and log link function rather than Two-part model as an initial descriptive evidence according to Buntin et al’s suggestion that one-part GLM model can avoid the problem of having to make post-hoc adjustment for heteroscedasticity to remove biases in predicted means and is easier to estimate than two-part OLS model[22]. In addition, it accommodates zero values without difficulty.

However, the result from GLM analyses cannot be causally interpreted due to several problems. First, although we controlled for a number of individual and household characteristics, there are still unmeasured attributes, which are likely correlated with the explanatory variables and medical expenditure simultaneously, leading to an omitted variable bias. Hence, we ran a standard static panel model and panel GLM model to tackle this matter. Hausman test was carried out to test the null hypothesis H_0_ of random effects against the alternative H_a_ of fixed effect.

Nonetheless, there are still other sources of bias to be addressed, one of which is an assumption that health status is static. While contemporaneous characteristics obviously affect medical expenditure, current medical expenditure also depends on medical expenditure from previous years because current health depends on previous health. Another concern is that, as noted by Dwyer et al(1999), causality between medical expenditure and working status may be bi-directional. That is, people with poor health status are less likely to engage in economic activity(14).

What has been the most commonly used in previous studies to address endogeneity due to reverse causality is Two Stage Least Squares (TSLS) with Instrumental Variable (IV). However, due to ongoing controversy on exogeneity of IV, we employed a dynamic panel Generalized Method of Moments (GMM) estimation with country fixed effect instead of TSLS. Serial correlation in the error terms, due to the panel nature of the dataset and the introduction of lagged variables, complicate the estimation procedure of the dynamic panel GLM model. Consequently, we applied a linear dynamic panel model, which allows the evaluation of unobserved heterogeneity and serial correlation of the error terms and is the most frequently used method in previous studies on health expenditure [23–25].

For an over-identified model like this, GMM is known to be an effective specification and hence has been used more commonly [26]. To better understand the dynamics of adjustment for medical expenditure, we specified the following dynamic function characterized by the presence of a lagged dependent variable among regressors;

$${ME}_{it}={\alpha ME}_{it-1}+{\beta job}_{it}+{\theta X}_{it}+s_{i}+\epsilon_{rt}$$

Where ME_it_ and ME_it-1_ are medical expenditure of *i* individual in *t* year and the previous year, respectively; job_it_ is working status of *i* individual; X_it_ is a vector of other control variables; and *s*_*i*_ is an individual-fixed effect.

First-difference GMM (Arellano—Bond GMM) forms moment conditions using lagged-levels of the dependent variable and the predetermined variables with first-differences of the disturbances [27]. However, it was found to perform poorly if the autoregressive process was too persistent (i.e., when α is close to unity) [28]. System GMM (Blundell–Bond GMM) exploits additional moment condition in which lagged differences of the dependent variables are orthogonal to levels of the disturbances, and thus can be a solution to this problem. However, System GMM requires initial conditions that the error term in the first period and the first-differenced exogenous variables are uncorrelated with the individual specific effect [29]. To address this issue, we estimated models with both specifications.

Once GMM estimators are obtained, conducting joint validity test of the instruments is a standard procedure [30]. First, the validity of over-identification restriction was verified with the Sargan-test that examined the null hypothesis, ‘all instruments chosen should not be correlated with residuals’. A failure to reject the null hypothesis implies that the instruments are valid. However, we should note that the rejection of Sargan-test does not necessarily mean that over-identification is not appropriate because the null hypothesis may also be rejected when error term does not meet “i.i.d (independent and identically distributed)” condition. Second, autocorrelation was also tested. Second-order autocorrelation should not be allowed in GMM estimation while the first-order autocorrelation can be.

For the outcome of unmet need, pooled logistic regression was performed as an exploratory analysis. Then, fixed effect panel logistic regression was employed to seek causality by controlling for unobserved systematic differences.

All the analyses were performed with Stata SE 14. For estimating Difference GMM and System GMM, we applied “xtabond2” and “xtdpdsys” respectively [31]. We also used “estat sargan” and “estat abond” to get the post-estimation specification tests.

## Results

### General descriptive results

After excluding observations aged less than 65 for the elderly group and less than 50 for the pre-elderly group and those missing any of the outcome and independent variables, the final analytical samples on medical expenditure and unmet needs included 20,451 and 14,170 elderly and 22,602 and 14,663 pre-elderly adults, respectively.

The distribution of medical expenditure and unmet needs according to the demographic and socioeconomic status for the elderly and the pre-elderly are shown in Table 1 and Table 2. Medical expenditure according to gender and education level showed opposite trends between the elderly and the pre-elderly groups. Among the elderly, males spent more on health service than females and so did those with a higher level of education than those with a lower level of education, but the opposite was found for the pre-elderly group. Households composed of a couple without a child showed the highest medical expenditure in both the elderly and the pre-elderly groups. Respondents without job, enrolled in health insurance, with disability, with household ownership, and chronic disease spent more than their counterparts.

**Table 1:** Descriptive statistics of medical expenditure and unmet need in the elderly.

Variable	Categories	Elderly					
		Medical expenditure			Unmet need		
		N	*Mean	SD	N	Lack of time	Financial burden
Gender	Male	8,871(43.4)	895	1,633	6,106(43.1)	1.8	6.7
	Female	11,580(56.6)	867	1,449	8,064(56.9)	2.1	10.1
Age	65 ≤ and <70	7,475(36.6)	852	1,442	4,625(32.6)	3.1	7.4
	70 ≤ and <75	6,442(31.5)	918	1,637	4,537(32.0)	2.3	9.0
	75 ≤ and <80	4,238(20.7)	910	1,466	3,060(21.6)	0.9	9.5
	80≤	2,296(11.2)	799	1,629	1,948(13.7)	0.4	9.2
Household type	Single	3,654(17.9)	818	1,332	2,634(18.6)	1.5	12.8
	Couple	9,538(46.6)	920	1,534	6,776(47.8)	2.2	6.8
	Couple + child	2,318(11.3)	887	1,703	1,592(11.2)	2.6	8.2
	Others	4,941(24.2)	841	1,578	3,168(22.4)	1.5	9.4
Education level	Lower than middle school	15,728(76.9)	855	1,488	10,803(76.2)	2.2	9.8
	High school graduate	3,219(15.7)	860	1,384	2,314(16.3)	1.4	5.9
	More than university	1,504(7.4)	1,172	2,131	1,053(7.4)	1.4	3.3
Job status	No	13,109(64.1)	937	1,616	9,010(63.6)	0.5	9.5
	Yes	7,342(35.9)	775	1,363	5,160(36.4)	4.5	7.2
Insurance type	Medicaid	1,801(8.8)	397	899	1,192(8.4)	0.8	14.6
	Health insurance	18,650(91.2)	925	1,572	12,978(91.6)	2.1	8.1
Income level	lowest	8,052(39.4)	751	1,317	5,627(39.7)	1.2	13.5
	Low	5,436(26.6)	919	1,537	3,782(26.7)	2.3	7.4
	Moderate	3,474(17.0)	955	1,601	2,411(17.0)	3.3	4.4
	High	2,042(10.0)	960	1,551	1,411(10.0)	2.1	3.8
	Highest	1,447(7.1)	1,147	2,212	939(6.6)	1.7	2.6
Disability	No	17,245(84.3)	853	1,481	11,934(84.2)	2.0	8.4
	Yes	3,206(15.7)	1,019	1,771	2,236(15.8)	1.7	10.1
Household ownership	Rent	5,077(24.8)	749	1,370	3,583(25.3)	1.4	13.2
	Ownership	15,374(75.2)	922	1,579	10,587(74.7)	2.2	7.1
Chronic disease	No	883(4.3)	384	1,173	472(3.3)	3.2	6.6
	Yes	19,568(95.7)	901	1,542	13,698(96.7)	1.9	8.7

*Unit: 1,000Korean won

**Table 2:** Descriptive statistics of medical expenditure and unmet need in the pre-elderly.

Variable	Categories	Pre-elderly					
		*Medical expenditure			Unmet need		
		N	Mean	SD	N	Lack of time	Financial burden
Gender	Male	10,745(47.5)	584	18	6,735(45.9)	5.4	4.4
	Female	11,857(52.5)	753	15	7,928(54.1)	6.1	6.3
Age	50 ≤ and <55	8,169(36.1)	564	22	5,148(35.1)	6.9	5.1
	55 ≤ and <60	7,477(33.1)	664	19	4,920(33.6)	5.6	5
	60 ≤ and <65	6,956(30.8)	808	18	4,595(31.3)	4.6	6.2
Household type	Single	1,276(5.6)	707	47	881(6.9)	7.2	10.1
	Couple	6,004(26.6)	767	23	3,916(26.7)	4.7	5.1
	Couple + child	11,269(49.9)	611	12	7,319(49.9)	5.6	3.9
	Others	4,053(17.9)	690	42	2,547(17.4)	7.3	8.6
Education level	Lower than middle school	11,049(48.9)	708	13	7,011(47.8)	6.5	7.3
	High school graduate	7,824(34.6)	653	19	5,199(35.5)	4.9	4.1
	More than university	3,729(16.5)	607	44	2,453(16.7)	5.5	2.7
Job status	No	6,974(30.9)	868	31	4,481(30.6)	7.7	4.6
	Yes	15,628(69.1)	585	10	10,182(69.4)	1.4	7.3
Insurance type	Medicaid	757(3.3)	389	36	487(3.3)	2.1	19.7
	Health insurance	21,845(96.7)	682	12	14,176(96.7)	5.9	4.9
Income level	lowest	2,300(10.2)	597	24	1,418(9.7)	4.1	14.2
	Low	4,356(19.3)	671	38	2,789(19.0)	5.5	9
	Moderate	5,033(22.3)	636	18	3,262(22.2)	6.7	5.5
	High	5,088(22.5)	671	19	3,300(22.5)	6.1	3.3
	Highest	5,825(25.8)	735	25	3,894(26.6)	5.5	1.4
Disability	No	20,996(92.9)	653	10	13,606(92.8)	5.9	4.9
	Yes	1,606(7.1)	924	95	1,057(7.2)	3.3	12.3
Household ownership	Rent	5,281(16.4)	599	18	3,545(24.2)	5.9	9.7
	Ownership	17,321(83.6)	694	14	11,118(75.8)	5.7	4
Chronic disease	No	3,708(16.4)	354	30	2,118(14.4)	6.4	3.6
	Yes	18,894(83.6)	735	13	12,545(85.6)	5.6	5.7

*Unit: 1,000Korean won

Generally, the prevalence of unmet need from lack of time was higher and the prevalence of unmet need from financial burden was lower in the pre-elderly than the elderly. The pattern of the prevalence of unmet needs according to sub-categories of independent variables was similar between two groups.

### Medical expenditure and unmet needs in the elderly

In Table 3 and Table 4, results from both the static (OLS and fixed effects) and dynamic panel models on medical expenditure in the elderly and the pre-elderly are presented. The result was very similar between the two GMM specifications. Medical expenditure responded to the employment status differently according to the modeling. Being employed was significantly associated with higher medical expenditure in both the static OLS and fixed-effects panel specifications, although coefficient attenuated after controlling for unobserved fixed characteristics. In the dynamic models, the lag of the dependent variable was statistically significant, which indicates some degree of persistence in the medical expenditure over time. Therefore, the result certainly rejects the static model in favor of the dynamic model.

**Table 3:** Job status and medical expenditure in the elderly: Cross-sectional and panel data analysis.

	Cross-sectional		Panel			
Variable	Pooled OLS	GLM	FE	Panel GLM	Arellano–Bond	Blundell—Bond
Observations	20,451	20,451	20,451	20,451	12,580	16,247
Lagged Medical expenditure					***0.00	***0.00
Job status						
Yes(Ref)						
No	***239,238	***223,584	*82,291	***198,450	-75,529	-87,827
(AR)(1) test					***-12.0	***-12.7
(AR)(2) test					-0.89	0.33
Sargan test					21.2	***45.3

*** p<0.001

** p<0.01

* p<0.05

**Table 4:** Job status and medical expenditure in the pre-elderly: Cross-sectional and panel data analysis.

	Cross-sectional		Panel			
Variable	Pooled OLS	GLM	FE	Panel GLM	Arellano–Bond	Blundell—Bond
Observations	62,498	22,602	62,498	22,602	12,840	17,233
Lagged Medical expenditure					***0.00	***0.00
Job status						
Yes(Ref)						
No	***223,732	***200,857	13,102	***180,640	**-196,462	**-194,287
(AR)(1) test					***-2.43	***-2.46
(AR)(2) test					1.60	2.06
Sargan test					***79.0	***149.5

*** p<0.001

** p<0.01

* p<0.05

The coefficient on the job status was negative and statistically non-significant in the dynamic model, indicating that job status had no association with medical expenditure in the elderly group. On the other hand, job status had an inverse and statistically significant association with medical expenditure at the 1% statistical significance level in the pre-elderly group.

In all the analyses for unmet need [Table 5], Hausman test rejected Ha, which is a strong indication of the validity of the fixed effects assumption. We found that unemployment was a significant determinant of lowering unmet need from lack of time while it was not significantly associated with unmet need from financial burden.

**Table 5:** Job status and unmet need in the elderly and the pre-elderly: Pooled logistic, FE.

Unmet Need	Variable	Time unmet need		Financial Unmet need	
		Pooled logistic	FE	Pooled logistic	FE
Elderly group	Job status				
	Yes (Ref)	Ref	Ref	Ref	Ref
	No	***0.13	**0.36	1.08	1.07
Pre-elderly group	Job status				
	Yes (Ref)	Ref	Ref	Ref	Ref
	No	***0.16	***0.38	*1.18	1.08

*** p<0.001

** p<0.01

* p<0.05

Table 6 shows the relevance of other potential correlates to the medical expenditure and unmet needs among the Korean elderly and pre-elderly adults. Household type and disability status were the only factors that were statistically significantly associated with medical expenditure in the elderly and the pre-elderly group, respectively. Contrary to previous documentations, we did not find a higher level of healthcare utilization in the Medicaid group compared to the health insurance group. As for unmet needs, age was a significant correlate with unmet need from lack of time in the elderly group and with unmet need from financial burden in the pre-elderly group. The elderly in moderate income level and the pre-elderly in high and highest income level were less likely to experience unmet need from financial burden compared to those in lowest income level.

**Table 6:** Result of the covariates.

	Elderly			Pre-elderly		
Variable	Medical expenditure	Unmet need(FE)		Medical expenditure	Unmet need(FE)	
		^†^(1)	^† †^ (2)		(1)	(2)
medical expenditure(-1)	***0.00			***0.00		
Gender						
Male	Ref	‒	‒	Ref	‒	‒
Female	442,507	‒	‒	305,736	‒	‒
Age						
65 ≤ and <70(50 ≤ and <55)	Ref			Ref		
70 ≤ and <75(55 ≤ and <60)	-73,132	0.96	0.92	57,801	0.76	**0.62
75 ≤ and <80(60 ≤ and <65)	-72,363	*0.46	1.07	135,346	0.83	***0.44
80≤		94,240	*0.07	0.93			
Household type						
Single	Ref			Ref		
Couple	*-232,032	1.02	0.79	105,215	0.60	1.85
Couple + child	-172,994	1.15	0.74	-15,579	0.89	2.26
Others	-117,636	1.25	0.86	-151,575	1.18	1.04
Education level						
Lower than middle school	Ref			Ref		
High school graduate	161,576	‒	1,288,616.86	55,373	0.00	150,916
More than university	629,201	‒	5.02	-152,061	0.00	-
Insurance type						
Medicaid	Ref			Ref		
Health insurance	174,546	1.33	1.74	338,108	2.73	*2.86
Income level						
Lowest	Ref			Ref		
Low	57,458	0.98	0.82	-16,087	0.88	0.88
Moderate	65,345	*1.87	**0.54	-7,758	1.20	0.68
High	110,466	1.51	0.64	-20,845	1.14	^†^0.63
Highest	65,005	0.67	0.74	47,783	1.20	*0.51
Disability						
No	Ref			Ref		
Yes	-154,034	1.84	*1.87	***-887,252	0.76	1.35
Household ownership						
Rent	Ref			Ref		
Ownership	64,619	1.45	0.74	-34,382	1.32	1.29
Chronic disease						
No	Ref			Ref		
Yes	33,706	3.86	0.83	-111,257	0.77	0.71

^†^ (1): unmet need du e to lack of time

^† †^ (2): unmet need due to financial matter

*** p<0.001

** p<0.01

* p<0.05

## Discussion

The association between employment status and health outcomes in the elderly has been the primary focus of the existing literature, and most studies reported that employment is beneficial for health of the elderly (4, 6–8). However, there has been a constant controversy on the potential for reverse causation given that healthier individuals are more likely to be employed for a longer time. At the same time, far less attention has been given to the effect of employment status on medical cost. Only two studies were found on the association between job status and medical expenditure in the elderly according to our literature search. Both of them used cross-sectional data, making them vulnerable to possibility of reverse causation [12, 13]. In addition, to our best knowledge there has been no evidence on the association between job status and medical expenditure in the pre-elderly adults, despite increasing concern about their financial vulnerability. This study attempted to identify the possible causal effect of job status on medical expenditure with consideration of unmet need together for the elderly and the pre-elderly population using a dynamic panel GMM model.

The first salient finding derived from this nationally representative, longitudinal sample based on methodology for addressing reverse causality was that job status is not a significant determinant of medical expenditure in the elderly, despite the widely-accepted proposition that employment is beneficial to health and hence reduce medical cost. Our finding was consistent with the result of a study by Shim Y (1997), which found no significant association between job status and health expenditure in the elderly living in Chung-buk province, Korea. On the other hand, Lim JY et al (2008) reported that participants in “Senior Employment Program” spent significantly less cost on health service compared to non-participants. By simply equating the level of medical expenditure with the level of health status, they concluded that employment enhanced participants’ health status. However, the level of medical expenditure does not necessarily translate to the level of health status since there are many factors that determine the utilization of health service among the elderly.

The result from our fixed-effect panel analysis regarding unmet need in the elderly indicated that not being engaged in economic activity has a strong association with lower odds of unmet need from lack of time, but has no significant association with unmet need from financial burden.

All the results taken together indicate that unemployment among the elderly does not have a significant effect on medical expenditure despite the unmet need from lack of time being significantly resolved. This suggests that delayed doctor visit from lack of time occurs only for trivial and low burden illnesses. In addition, since the elderly over 65 years of age are eligible for a national pension, which is the biggest financial safety net for the elderly in Korea, they might be at a relatively low risk for under-utilization of medical service caused by financial constraint.

On the other hand, rather concerning results were found for the pre-elderly group. We observed a phenomenon that we had initially thought would occur in the elderly group: unemployment was significantly associated with lower medical expenditure while unmet needs from financial burden did not significantly change among the pre-elderly adults. This may indicate that lower medical expenditure among the unemployed pre-elderly adults is not due to improved health but rather from a reduction of medical service utilization from decreased income.

In Korea, the current pre-elderly group aged 50 to 65 composes of a “baby boomer” generation born right after the Korean War. This age group represents a phase of life when various health problems begin to arise. Many of the pre-elderly adults have already retired from their jobs or are at great risk for early involuntary retirement while they are still ineligible for the pension benefits. Since security net for this generation is not enough with much room for progress, they are highly likely to fall into poverty as a result of increasing medical expenditure, decreasing income level, and being ineligible for the old age pension. Accordingly, being unemployed may hit the pre-elderly harder than the elderly in terms of economic hardship, and refrain the unemployed pre-elderly adults from utilizing proper medical service when they fall ill. According to the behavioral model developed by Ron Andersen in 1968, the use of health service is a function of the predisposing, enabling, and need characteristics of the individual[32]. Employment status might have an effect on healthcare utilization mainly through the mechanism of changing the enabling factors such as income and availability of time to visit doctors.

The contrasting result between the pre-elderly and the elderly groups might be partly due to different composition of job positions. When we disaggregated the employed elderly and pre-elderly by 9 kinds of job positions (permanent, regular, temporary, daily worker, workers in government job placement project, self-employer, non-paid workers in family business, employer, non- applicable), the share of respondents in the “non-applicable” category was large in both groups, but higher in the elderly than in the pre-elderly adults (pre-elderly: 36.7% vs elderly: 58.9%). The non-applicable category includes all kinds of employment in the informal sector for which payment is very low. This suggests that the pre-elderly adults tend to engage in more formal and better-paid jobs, while the elderly work for informal and inconsequential jobs. Therefore, losing or quitting jobs may have a bigger influence on the expenditure pattern for the pre-elderly adults than for the elderly group who are already subsisting on a very small income even when they are employed. In addition, the share of respondents working in permanent jobs was higher among the pre-elderly than the elderly (pre-elderly: 9.5% vs. elderly: 0.4%). Considering that permanent job positions are generally the most secure type of employment, the large number of pre-elderly adults who transit from employed to unemployed status may feel very insecure and stressed out, which make them refrain from spending.

Delaying or withholding utilization of health service despite persisting unmet needs can be linked to worsening health status. Unemployment among the pre-elderly adults may be taken less seriously based on the common conception that they have more willingness and capacity to find a job again than the elderly. However, even a temporary delay in the use of health service can bring about substantial consequences because for some diseases failing to detect in time can cause irreparable health outcomes. Even if the health consequence is not fatal, delaying the use of health service still causes a vicious cycle of worsening health, which again decrease chance of returning to the job market. Therefore, creating and expanding safety nets to protect the unemployed pre-elderly deserves further attention from the aging societies.

## Conclusions

Much of the responsibility for care of the elderly has been shifted from the private to the public sector, which led to an expansion of social security system for the elderly. Yet, implications for the pre-elderly population have been largely unexplored. This paper shed a light on the need to pay urgent attention to the impact of unemployment on health utilization behavior among the pre-elderly adults. The pre-elderly “baby boomers” in Korea stand at a crossroad facing double responsibilities to support their children’s education and care for their parents. Many of them are not fully ready for their post-retirement life yet, and unfortunately they will be the first target group to be sacrificed when income declines [33]. Our estimates revealed that unemployed pre-elderly group is more likely to reduce their medical spending. The policy dialogue on safety net currently centers only on the elderly, but our study suggests that it should be extended to the pre-elderly population, especially those who are unemployed.
